# Reversible posterior leukoencephalopathy syndrome following apatinib for gastric cancer in an adult: A case report and a review of the literature

**DOI:** 10.1097/MD.0000000000017787

**Published:** 2019-11-15

**Authors:** Yajuan Lv, Yan Zhang, Jiandong Zhang, Ning Liang, Fengjun Liu, Ruixue Liu

**Affiliations:** a Department of Radiotherapy,The First Affiliated Hospital of Shandong First Medical University, Shandong; b Weifang Medical University, P.R. China.

**Keywords:** apatinib, case report, gastric cancer, reversible posterior leukoencephalopathy syndrome

## Abstract

**Rationale:**

Reversible posterior leukoencephalopathy syndrome (RPLS) is characterized by rapidly progressive hypertension, headache, and disturbance of consciousness. Moreover, RPLS is rarely reported after apatinib treatment.

**Patient concerns:**

We present a case of RPLS induced by apatinib in this report. The patient had dizziness and bilateral lower limb weakness after apatinib use for 12 days.

**Diagnosis and interventions:**

Cranial T2-weighted magnetic resonance imaging (MRI) revealed symmetrical increased signal intensity in bilateral areas of the basal ganglia, radiation crown, frontal lobe, parietal lobe, and occipital lobe, which was suggestive of RPLS. The patient discontinued apatinib use and was administered dexamethasone, mannitol, and antihypertensive drugs.

**Outcomes:**

The patient's blood pressure returned to normal and neurological symptoms improved after 3 days of discontinuation of apatinib use. Moreover, brain MRI showed complete resolution of previous changes after 44 days of discontinuation of apatinib use.

**Lessons:**

Increased blood pressure may damage the normal blood-brain barrier, resulting in the extravasation of the fluid into the brain parenchyma. Hypertension is a significant cause of RPLS. It is important to strictly monitor blood pressure during apatinib treatment.

## 1 Introduction

Reversible posterior leukoencephalopathy syndrome (RPLS) is characterized by rapidly progressive hypertension, headache, and disturbance of consciousness, which was first reported by Hinchey et al.^[1]^ Most cases of RPLS have usually acute onset, which can develop in both children and adults.^[2,3]^ There are many causes of RPLS, the most common causes are hypertensive encephalopathy, eclampsia, and chemotherapy in patients with malignant tumors.^[2]^ The typical symptom of RPLS is bilateral symmetric vasogenic edema in the posterior subcortex of the brain.^[2,3]^ Cranial computed tomography (CT) and magnetic resonance imaging (MRI) are commonly used in diagnosis of RPLS. The lesions usually have a low density on CT, an equal or low signal on T1 weighted imaging (T1WI), a high signal on T2 weighted imaging (T2WI), and a high signal on fluid-attenuated inversion recovery MRI.^[2,3]^ Most patients can usually achieve full recovery without any abnormal neurological symptoms. Gastric cancer (GC) is one of the most common malignancies worldwide, and approximately 41% of patients with GC come from China.^[4]^ Apatinib is an anti-angiogenic targeting drug that was proven safe and effective for patients with advanced GC after failure of standard chemotherapy. The common side effects of apatinib are hematological toxicity, hypertension, hand-foot syndrome, and gastrointestinal reactions.^[5]^ A case report previously demonstrated RPLS caused by apatinib use, but the patient had metastatic cervical cancer and RPLS developed 3 months after application of apatinib.^[6]^ We report a case of RPLS caused by apatinib use for metastatic GC, and RPLS developed only 12 days after initiation of apatinib treatment.

## 2 Case presentation

A 56-year-old man with metastatic GC and was admitted to our hospital due to “dizziness and bilateral lower limb weakness.”

The patient had a history of type 2 diabetes for 6 years and had no history of other chronic diseases such as hypertension. He was initially diagnosed with stage IV GC in April 2012. He underwent several many cycles of chemotherapy due to disease progression. The last cycle of chemotherapy (paclitaxel liposome combined with epirubicin) was performed on May 10, 2018. The patient underwent palliative radiotherapy due to right scapular metastasis and right adrenal metastasis in April 2016 and March 2017, respectively, with no apparent discomfort. From the date of diagnosis of GC until June 2018, the patient had not used any targeted drugs and had normal blood pressure.

**Figure 1:**
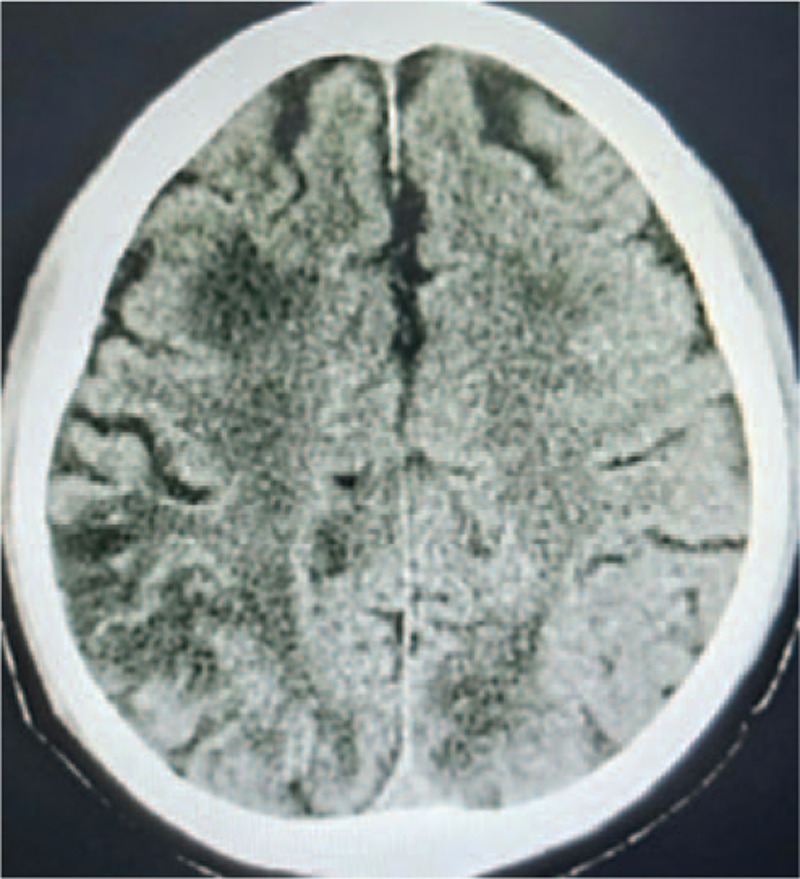
Brain computed tomography (CT) image on August 1, 2018.

**Figure 2:**
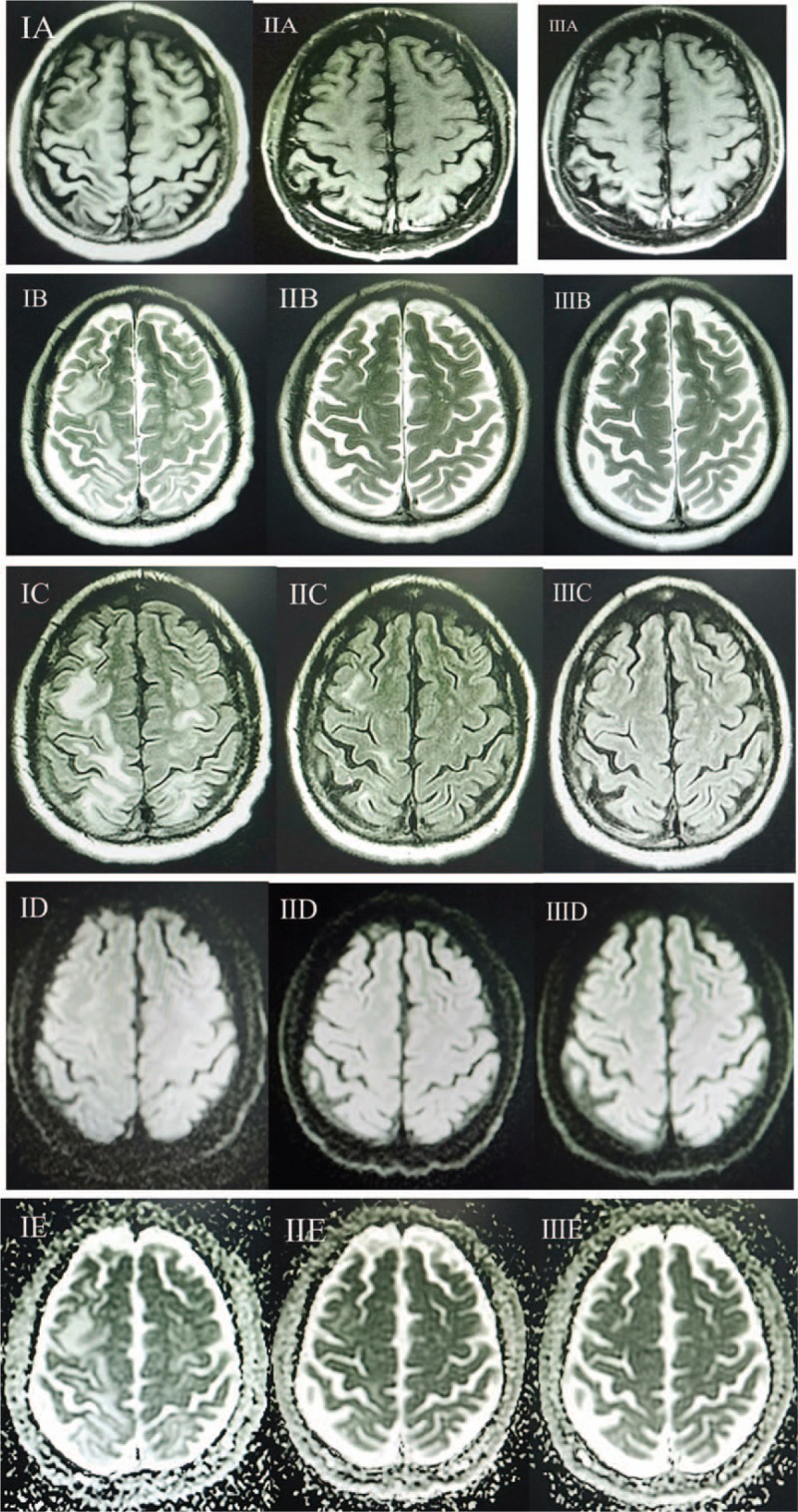
Brain magnetic resonance imaging (MRI). I, Brain MRI scan on August 2, 2018. II, Brain MRI scan on August 13, 2018. III, Brain MRI scan on September 5, 2018, respectively. A, MRI T1 FLAIR, B, MRI T2, C, MRI T2 FLAIR, D, DWI, E, ADC, respectively. ADC = apparent diffusion coefficient, DWI = diffusion-weighted imaging, FLAIR = fluid-attenuated inversion recovery.

The patient was admitted to our department for review on July 16, 2018. A CT scan showed that the left adrenal metastasis was significantly greater than before. The patient refused to undergo chemotherapy. He had a Karnofsky (KPS) score of 100. Hematological indicators, including liver function, kidney function, blood cell analysis, electrolytes, and coagulation index were normal. Blood pressure was also in normal range. He was advised to use apatinib (750 mg p.o. q.d.) on July 19, 2018. He had no obvious discomforts for 7 days after oral apatinib administration, and he was discharged. Blood pressure was normal during hospitalization. The patient was advised to monitor blood pressure by daily at home, but he did not perform it. He had dizziness accompanied by weakness in both lower limbs on the evening of July 31, 2018 (12 days after oral apatinib administration). Blood pressure was 185/110 mm Hg. He was administered valsartan 80 mg. There were no any changes in his blood pressure measured 2 hours after administration of this drug, and he went to sleep. The patient was admitted in our department on the morning of August 1, 2018. Blood pressure after admission was 198/111 mm Hg. He did not use any other drugs that may cause an increase in blood pressure at home. He was instructed to discontinue apatinib use. Nifedipine controlled-release tablet 20 mg and furosemide 20 mg were administered immediately. Blood pressure was reduced to the normal range in 4 hours after drug administration. Hematological indicators, such as liver function, kidney function, blood cell analysis, electrolytes, etc were normal. Seizures, however, occurred twice during this period, and his consciousness improved after using diazepam. The patient reported vision loss on both eyes after the second seizure occurrence. Physical examination revealed that the bilateral eyeballs squinted to the left side, the size of both pupils were normal, and light reflection on both eyes was slightly dull. Brain CT scan on the afternoon of August 1 revealed bilateral basal ganglia, radial crown, frontal lobe, parietal lobe, and occipital lobe with patchy low-density areas (Fig. 1). On August 2, cranial T2-weighted MRI revealed symmetrical increased signal intensity in bilateral areas of the basal ganglia, radiation crown, frontal lobe, parietal lobe, and occipital lobe, which was suggestive of RPLS (Fig. 2-I). Diffusion-weighted imaging showed no obvious abnormal signal (Fig. 2-ID). Mannitol [150 mL intravenous (i.v.) drip q12 h], dexamethasone (5 mg i.v. q.d.), and phenobarbital sodium (0.1 g i.m. q8h) were administered to him. Blood pressure was maintained at 97 to 126/59 to 75 mm Hg, and antihypertensive drugs were discontinued. No seizure occurred, vision returned to normal, and bilateral lower limb weakness improved 3 days after treatment. A repeat MRI scan of the brain on 13th August showed partial resolution of previous the abnormalities (Fig. 2-II). The patient was discharged on 20th August. A brain MRI scan on 5th September showed complete resolution of previous changes (Fig. 2-II). Without using any antihypertensive drugs anymore, his blood pressure remained normal until now (April 25, 2019). Moreover, the patient has no obvious signs of discomfort and can take care of himself until the follow-up (by phone on April 25, 2019).

## 3 Discussion

RPLS is a rare acute neurological clinical syndrome, and can be caused by chemotherapy therapy and targeted therapy in patients with cancer.^[2]^ RPLS is usually associated with many risk factors such as hypertension and renal failure.^[3]^ Anti-angiogenic drugs are widely used in cancer therapy, but these drugs have several adverse effects, such as hypertension and hemorrhage.^[7]^ RPLS is, however, a rare side effect of anti-angiogenic drugs, which is commonly reported in cases.^[7]^ Bevacizumab and sunitinib are the most common anti-angiogenic drugs, which can induce RPLS.^[7,8]^ From 2006 to 2016, a total of 22 cases of RPLS following bevacizumab treatment from “PubMed” database were reported. Among these patients, 20 cases were resolved after withdrawal of bevacizumab and strict control of blood pressure, whereas 2 cases resulted to death. Costa et al reported 8 cases with RPLS following sunitinib therapy. The time of RPLS development after sunitinib use was different, ranging from 1 week to 8 months. Patients received symptomatic treatment. Seven cases got complete response (CR) and 1 case got partial response.^[9]^ Other rare antivascular endothelial growth factor receptor (VEGFR) drugs in addition to bevacizumab and sunitinib that can cause RPLS are shown in Table 1. A total of 14 patients developed RPLS within 3 months after drug administration, and 13 patients had increased blood pressure (Table 1). After discontinuing anti-VEGFR drug treatment and receiving symptomatic treatment, 12 patients achieved CR, but 1 patient died.^[6,10–21]^

**Table 1:**
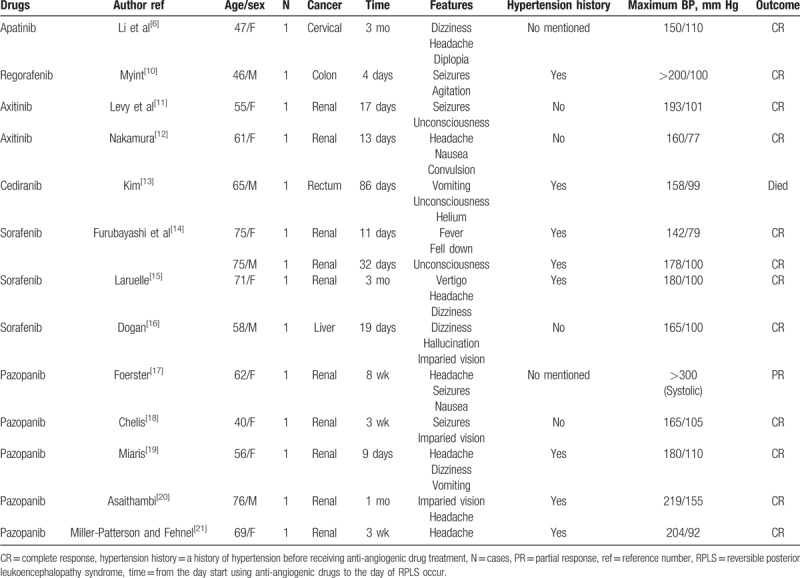
Case reports of RPLS associated with VEGFR inhibitors.

Apatinib, a small molecule tyrosine kinase inhibitor that selectively binds to VEGFR-2, was developed in China and was indicated for advanced and metastatic GC.^[5]^ A phase III trial showed that apatinib rather than placebo significantly improved overall survival and progression-free survival (PFS) in patients with advanced GC after 2 lines of previous chemotherapy. Moreover, the common grade 3 to 4 nonhematological adverse events were hypertension, hand-foot syndrome, and proteinuria.^[22]^ As reported in a meta-analysis of 1069 patients with advanced GC demonstrating that apatinib shows short-term efficacy than placebo regardless of its use as first- or second-line chemotherapy. RPLS has not been reported in this review.^[23]^ RPLS is a rare side effect of apatinib therapy. There are various symptoms of RPLS, such as headache, lethargy, memory loss, abnormal vision, epilepsy, etc. It is important to note that none of these symptoms are necessary in the diagnosis of RPLS. A case of RPLS with apatinib therapy was reported in a patient with metastatic cervical cancer.^[6]^ The patient developed headache, dizziness, and blurred vision after administration of apatinib for 3 months (500 mg p.o. q.d.). She received symptomatic treatment, and her symptoms significantly improved after 1 week. Our patient had no symptom of headache, which is different from that was previously reported.^[6]^ Our patient showed a significant increase in blood pressure after apatinib administration for only 12 days. This patient had acute onset, typical MRI lesions, and did not use any other drug before the onset of RPLS. Clinical symptoms disappeared after discontinuation of apatinib use. These were the supporting evidences that apatinib use was the cause of RPLS. In addition, the patient had seizures twice on the first day of admission. This symptom has not been previously reported in another case report.^[6]^

The pathogenesis of RPLS of anti-angiogenic drugs is unclear. Tam et al^[24]^ had reported that there were 3 risk factors that may induce RPLS, including significant fluid overload (>10% baseline weight), mean blood pressure >25% of baseline, and creatinine >0.16 mmol/L. Our patient only had high blood pressure. It met one of the Tam et al's^[24]^ risk criteria (mean blood pressure >30% of baseline).^[24]^ It has been proposed that targeted anti-VEGFR drugs can cause blood-brain barrier changes by damaging vascular endothelial cells. In addition, hypertension caused by the anti-VEGFR drugs will damage the normal blood-brain barrier, resulting in extravasation of the fluid in to the brain parenchyma.^[7,25]^ Cerebrovascular spasm may cause hypothalamic ischemia and hypoxia may also be another mechanism.^[26]^ The mechanism that causes RPLS still needs further research.

The incidence of RPLS is extremely low and needs to be differentiated from those of brain metastasis and stroke. It is necessary to perform brain MRI after symptomatic therapy to avoid misdiagnosis. The primary treatment is to immediately discontinue the use of anti-VEGFR drug. Some drugs such as mannitol, dexamethasone, and antihypertensive drugs should be immediately administered to patients to reduce brain edema, otherwise it may endanger the patient's life. In addition, blood pressure needs continuous monitoring, and the doses of antihypertensive drugs should be adjusted according to the blood pressure. Blood pressure of most patients would return to normal value after discontinuing the use of anti-angiogenic drugs. The prognosis of the majority of cases is good.^[2]^ RPLS is usually accompanied by hypertension. Strict monitoring of blood pressure is necessary during the administration of VEGFR inhibitors.^[7]^

## Author contributions

**Data curation:** Ruixue Liu.

**Resources:** Ning Liang, Fengjun Liu.

**Writing – original draft:** Yajuan Lv, Yan Zhang.

**Writing – review and editing:** Jiandong Zhang.

Jiandong Zhang orcid: 0000-0003-0169-7092.
